# Seroprevalence of *Neospora caninum* in local Bali dog

**DOI:** 10.14202/vetworld.2018.926-929

**Published:** 2018-07-14

**Authors:** I Made Dwinata, Ida Bagus Made Oka, Kadek Karang Agustina, I Made Damriyasa

**Affiliations:** 1 Department of Parasitology, Faculty of Veterinary Medicine, Udayana University, PB. Sudirman St. Campus, Denpasar, Bali 80223, Indonesia; 2 Department of Public Health, Faculty of Veterinary Medicine, Udayana University, PB. Sudirman St. Campus, Denpasar, Bali 80223, Indonesia; 3 Department of Clinical Pathology and Diagnosis, Faculty of Veterinary Medicine, Udayana University, PB. Sudirman St. Campus, Denpasar, Bali 80223, Indonesia

**Keywords:** local Bali dog, *Neospora caninum*, seroprevalence

## Abstract

**Aim:**

The aim of this research was to identify the seroprevalence of *Neospora caninum* in local Bali dogs.

**Materials and Methods:**

A total of 147 local Bali dog serum samples have been examined for antibodies of *N. caninum* using enzyme-linked immunosorbent assay test.

**Results:**

The results confirmed that 5 (3.4%) local Bali dogs have antibody for *N. caninum*. There were no significant differences in seroprevalence of *Neospora* infection in local Bali dogs between different genders, ages, and take care methods (p>0.05).

**Conclusion:**

The results provided evidence for the presence of *N. caninum* infection in local Bali dogs and thus the risk to Bali cattle and dog health.

## Introduction

*Neospora caninum* is an *apicomplexan* protozoon that can infect dogs, cattle, and other mammalians [1,2]. Dogs are well known as a definitive host and can spread oocyst [3,4]. Dogs get infection by oral transmissions and transplacental [5,6]. Severe *Neosporosis* usually occurs in congenital cases. It causes neuromuscular symptoms with paralysis of the legs and leads to the lethal encephalomyelitis [7,8]. *N. caninum* has been regarded as the major cause of abortion with great economic impact in cattle industry [9-12]. Prevention and control of *Neosporosis* in cattle are necessary, especially, if there is a role of wildlife in its transmission [13-16]. The seroprevalence of *N. caninu*m has been reported to infect dogs in many countries, such as 20.9% of prevalence in farm and kennel dogs in Southern Italy [17], 31% in stray dogs in Iran [18], 55.5% of prevalence in Turkey [19], and 15% of prevalence in China [20], but there are no data reported in Indonesia.

Serological results in multiple species, including domestic, wildlife, and zoo animals, provide evidence that many species have been exposed to this parasite [12]. Nowadays, data about Neospora infection in animals in Asia, particularly, in Indonesia are limited. There was a study reported that *Neosporosis* in Bali cattle was 5.5% in Bali. It is believed to cause the reproductive disorder that occurs in Bali cattle in Bali [21]. While the seroprevalence of *Neosporosi*s in cattle has been reported 44.9% in Taiwan [22] and 9% in Malaysia on dairy farms [23]. Recent studies reported a high seroprevalence of cattle *Neosporosis*, i.e., 43% in Pakistan [24], 37.7% in Turkey [19], and 46.9% in Northern Provinces of Thailand [25].

In Bali, the pattern of cattle productions is generally traditional [26,27]; this situation allows free roaming dogs to access the cowshed, interact, and spreading agents. Therefore, it is necessary to study the epidemiology of *Neosporosis* in local Bali dogs in Bali to reveal the role of dogs as a source of transmission of *Neosporosis* in Bali cattle.

## Materials and Methods

### Ethical approval

This study was approved by the ethics commission for the use of animal in the research and education of The Faculty of Veterinary Medicine Udayana University and declared eligible with reference No. 275a/KE-PH/XII/2016.

### Samples

The sample size was calculated using the formula proposed by Thrusfield [28]. A total of 147 local Bali dog serum samples have collected from Bali, Indonesia. Each sample is grouped by gender, age, and take care methods, i.e., 108 males and 39 females, 87 <1 year old and 80 more than 1 year old, and 77 caged and 70 free roam, respectively. Dog samples were targeted in local Bali dogs that live around the Bali cattle farm.

### Preparation of the serum samples

Blood sampling was performed in eight regencies located in the study area. After the dogs had fasted overnight, 5 ml of blood was taken from the cephalic vein and collected into vacuum tubes without an anticoagulant agent. Sera were obtained by centrifugation for 10 min at 358 g and stored at −20°C until assayed [29] at the Department of Parasitology, Faculty of Veterinary Medicine, Udayana University.

### Enzyme-linked immunosorbent assay (ELISA)

The presence of antibodies to *N. caninum* was detected by indirect ELISA using a commercially available antigen of *N. caninum* (ID.Vet^®^, ID Screen *N. caninum* Indirect Multi-species). The kit procedure is based on a solid-phase indirect ELISA. A positive result is indicated by the development of a blue color. The reaction is stopped by adding of the stop solution; the color changes to yellow. The result is read by a microplate photometer, where the optical density is measured at 450 nm [30].

### Statistical analysis

Seroprevalence was statistically analyzed, considering the variables of gender, age, and take care methods. The data analysis was performed with Pearson Chi-square test using SPSS 17.0, which the differences were considered statistically significant when p≤0.05.

## Results and Discussion

This is the first report about *N. caninum* seroprevalence in dogs in Indonesia. We found that the seroprevalence of *N. caninum* in local Bali dogs was 3.4% (5/147). This data is supporting the previous report that found seroprevalence of *N. caninu*m in Bali cattle in the area of study [21], indicates that there is a relationship between Neosporosis in dogs and Bali cattle in Bali.

This result is in agreement to previous studies that found 6.4% of seroprevalence of *N. caninum* in dogs using indirect fluorescent antibody test in Italy [31], 8.3% in Amazon, Brazil [32], and 6.25% in Jilin, China using Neospora agglutination test [33].

This finding is lower than the seroprevalence of dog’s Neosporosis in some previous studies, i.e., 15% in Central China [20], 16.36% in Poland [34], and 31% in Iran [18]. The differences in seroprevalence of *N. caninum* in dogs in some countries indicate that there are the factors that affected the result such as type of dog, geo and topography, population, management system, and examination methods used [35-37]. Low prevalence of *N. caninum* in local Bali dogs is probably due to environmental factors that are less suitable for oocyst development. Transmission is mostly from dog to dog by eating sporulated oocyst [1], and rarely found dogs eat aborted cattle placenta in Bali. Season factor greatly influences the occurrence of Neosporosis [38], where this research was carried out in the dry season with hot temperatures that can disrupt the development of oocysts into an infective stage. In addition, the low number of oocysts released by natural *N. caninum-*infected dogs [4].

The presence of free-roaming dogs in Bali was so many [39]. With the detection of *N. caninum* antibodies in local Bali dogs will be a considerable threat to Bali cattle. The infection happens considering the cattle rearing system in Bali are still with semi-intensive and traditional [26,27]. Dog and Bali cattle have a close interaction that dogs can easily access the cattle house.

Based on the variables observed in this study (Table-1), all factors, i.e., gender, age, and upkeep methods were not significantly affected the seroprevalence of *N. caninu*m in local Bali dogs (p>0.05). Regarding the dog gender, we found that 2.8% (3/108) positive male dogs and 5.1% (2/39) positive female dogs. This data is in agreement with the previous report [20,40]. However, a study in 2011 found a higher prevalence in female dogs than male dogs [37]. Related to the ages of dogs, the result shows that the seroprevalence of under 1 years old dogs was 2.3% while 5% in dogs of upper 1 year of age. This finding is similar to the report in Italy [31] and Pakistan [24], and there was no age effect on the magnitude of *N. caninum* seroprevalence. However, in contrary to the statement that *N. caninum* infections have a correlation to the age of dogs, young dogs were more vulnerable than adult dogs [41]. According to the upkeep methods, the seroprevalence of *N. caninum* on caged and free-roaming dogs was, respectively, 1.3% and 5.7%. The similar result reported in Brazil [42] but slightly different in Italy, the seroprevalence of Neosporosis in stray dogs was 35.8% higher than caged dogs which was 17.3% [17]. This is explained that free-roaming dogs are more likely to contact with the source of infection either from the infective *N. caninum* oocyst or the aborted cattle placenta.

**Table-1:** Seroprevalence of *N. caninum* in Bali local dogs in Bali.

Variable	Sample	Infected	(%)	*χ*^2^	p value
Gender					
Male	108	3	2.8	0.488	0.609^ns^
Female	39	2	5.1		
Age					
≤1 year old	87	2	2.3	0.789	0.399^ns^
>1 years old	60	3	5.0		
Upkeep method					
Caged	77	1	1.3	2.176	0.192^ns^
Free roam	70	4	5.7		
Total	147	5	3.4		

*ns=Not significant, *N. caninum=Neospora caninum*

## Conclusion

The seroprevalence of *N. caninum* in local Bali dogs was 3.4%. Gender, ages, and take care methods were not affected the prevalence of this parasite.

## Authors’ Contributions

IMD participated in the fieldwork, sample collection, laboratory work, ELISA test, and manuscript drafting; IBMO participated in the fieldwork, sample collection, laboratory work, and manuscript drafting; KKA participated in the fieldwork and participated in manuscript drafting; IMD designed the research and participated in manuscript drafting. All authors read and approved the final manuscript.
